# Association of Copy Number Variation Signature and Survival in Patients With Serous Ovarian Cancer

**DOI:** 10.1001/jamanetworkopen.2021.14162

**Published:** 2021-06-28

**Authors:** Ryon P. Graf, Ramez Eskander, Leo Brueggeman, Dwayne G. Stupack

**Affiliations:** 1 Moores Cancer Center, University of California, San Diego; 2 Interdisciplinary Genetics Program, University of Iowa, Iowa City; 3 Medical Scientist Training Program, University of Iowa, Iowa City; 4 Now at Foundation Medicine Inc, Cambridge, Massachusetts

## Abstract

**Importance:**

Tailoring therapeutic regimens to individual patients with ovarian cancer is informed by severity of disease using a variety of clinicopathologic indicators. Although DNA repair variations are increasingly used for therapy selection in ovarian cancer, molecular features are not widely used for general assessment of patient prognosis and disease severity.

**Objective:**

To distill a highly dynamic characteristic, signature of copy number variations (CNV), into a risk score that could be easily validated analytically or repurposed for use given existing US Food and Drug Administration (FDA)–approved multigene assays.

**Design, Setting, and Participants:**

This genetic association study used the Cancer Genome Atlas Ovarian Cancer database to assess for genome-wide survival associations agnostic to gene function. Regions enriched for significant associations were compared to associations from scrambled data. CNV associations were condensed into a risk score, which was internally validated using bootstrapping. The participants were patients with serous ovarian cancer (stages I-IV) diagnosed from 1992 to 2013. Statistical analysis was performed from April to July 2020.

**Main Outcomes and Measures:**

Overall survival (OS).

**Results:**

Among 564 patients with serous ovarian cancer, the mean (SD) age was 59.7 (11.5) years; 34 (6%) identified as Black or African American. A total of 13 genome regions, comprising 14 alterations, were identified as significantly risk associated. Composite risk score was independent of total CNV burden, total mutational burden, *BRCA* status, and open-source genome-wide DNA repair deficiency signatures. Binned terciles yielded high-, standard-, and low-risk groups with respective median OS estimates of 2.9 (95% CI, 2.3-3.2) years, 4.1 (95% CI, 3.7-4.8) years, and 5.7 (95% CI, 4.7-7.4) years, respectively (*P* < .001). Associated 5-year survival estimates in each tercile were 15% (95% CI, 10%-22%), 36% (95% CI, 29%-46%), and 53% (95% CI, 45%-62%). The risk score had more discriminatory ability to prognosticate OS than age, clinical stage, grade, and race combined, and was strongly additive to significant clinical features (*P* < .001). Simulated adaptation of FDA-approved assays showed similar performance. Gene ontology analyses of identified regions showed an enrichment for regulatory miRNAs and protein kinase regulators.

**Conclusions and Relevance:**

This study found that a CNV-based risk score is independent to and stronger than current or near-future ovarian cancer genomic biomarkers to prognosticate OS. CNV regions identified were not strongly associated with canonical ovarian cancer biological pathways, identifying candidates for future mechanistic investigations. External validation of the CNV risk score, especially in concert with more extensive clinical features, could be pursued via existing FDA-approved assays.

## Key Points

**Question:**

Do copy number variation (CNV) signatures inform prognosis for patients with ovarian cancer?

**Findings:**

In this genetic association study of 564 patients with serous ovarian cancer, an internally validated CNV signature from The Cancer Genome Atlas had more discriminatory ability to prognosticate overall survival than age, clinical stage, grade, and race combined, as well as gross CNV burden, total mutational burden, *BRCA* status, and open-source candidate genome-wide DNA repair deficiency signatures.

**Meaning:**

These findings suggest that a CNV-based risk score is independent to and stronger than current or near-future ovarian cancer genomic biomarkers, as well as available clinical features, to prognosticate survival.

## Teaser

This genetic association study examines the Cancer Genome Atlas database to assess for genome-wide survival associations agnostic to gene function among patients with serous ovarian cancer.

## Introduction

Ovarian cancer remains the most lethal gynecologic malignancy, with an estimated 21 750 new cases and 13 940 deaths predicted in the United States for 2020.^1^ Despite notable recent advancements in the use of PARP inhibitors, particularly as maintenance therapy^2,3,4^ (neo)adjuvant combination platinum-based chemotherapy regimens and surgical cytoreduction have remained frontline treatment for newly diagnosed ovarian cancer for the last 40 years. Breast cancer gene 1 (*BRCA1*) and *BRCA2* variations (germline and somatic) have been reported in up to 20% of patients with high grade serous ovarian cancer, with nearly 50% harboring homologous recombination deficiency (HRD), both associated with significant clinical benefit to PARP inhibitor therapy.^2,3,4,5,6^ Beyond this, clinical approaches to managing ovarian cancer have not broadly benefited from precision medicine. A range of decisions are required to tailor therapeutic and follow-up regimens to individual patients, all of which are directly or indirectly informed by the general prognosis. The ability to prognosticate survival with standard of care in this patient population, especially long-term survival, is useful to guide therapeutic choices as well as patient follow-up and monitoring.

A number of risk scores and nomograms have been developed to help consolidate and prognosticate ovarian cancer patient risk of death over time. Most clinically used nomograms focus strongly on clinicopathological features.^7,8,9^ Although inexpensive and ubiquitous, clinical features such as performance status are prone to degrees of subjectivity and considerable interoperator discordance.^10,11^ It has been suggested that risk assessments that incorporate genomic features might add accuracy and robustness.

Prognostic models incorporating variable genomic parameters have been forwarded to better predict long-term survival in ovarian cancer. Some of these outperform models based solely upon clinicopathological features,^12,13,14^ but most have not been widely adopted for use because of the complex laboratory infrastructure required. Translating a molecular model or signature to clinical use requires robust, scalable, and readily available diagnostics. The development and implementation of any molecular diagnostics involves a tradeoff between scalability, complexity, and cost.

Different cancer types display varying degrees of copy number variations (CNV), for which serous ovarian cancer has a wide spectrum and diversity of alterations.^15,16^ CNV assessments can be readily performed on tumor samples, even those that are formalin-fixed paraffin-embedded (FFPE)–derived DNA, using low coverage whole genome sequencing (<20×, <2× reported) with low cost and bioinformatic load.^17,18^ These approaches are robust relative to protein and mRNA, which require greater preanalytic handling and care. Importantly, a growing number of health care insurers are reimbursing commercially available hybrid capture multigene assays, which both incorporate CNV information and are approved by the FDA.

Given these factors, we were motivated to explore the potential for a signature risk score using CNV that was additive to existing clinicopathological features. Critically, such an approach had to be amenable to the use of simple, cost-effective and/or available and reimbursed molecular diagnostic technology.

## Methods

A flowchart of the approach to analyses is provided as eFigure 1 in the Supplement. All specimens were obtained from patients with appropriate consent from the relevant institutional review board. As this study was conducted using publicly available and deidentified data, it did not require institutional review board approval or informed patient consent, in accordance with 45 CFR §46.

### Data

The TCGA Firehose Ovarian Cancer data set contains both genomic and clinical outcomes data,^19^ including both genome-wide copy number assessments for more than 20 000 genes and overall survival data for nearly 600 patients with ovarian cancer, some with more than 10 years of follow-up. Race/ethnicity data were self-reported. The TCGA Firehose database was last accessed on July 2, 2020; analyses reflected these data. CNV data used the provided Genomic Identification of Significant Targets in Cancer (GISTIC)^20^ calls directly without further tuning.

### Statistical Analysis

#### Model Building

Because it cannot be assumed that a degree of gain or loss of copy number of a gene will have a linear relationship with expression, nor that the linear scale would carry an approximate normal distribution from gene to gene, gene gains and losses were assessed separately as individual dichotomous variables. A GISTIC call of +1 or +2 was considered a gain and −1 or −2 was considered a loss. The patterns of gene gains and losses in ovarian cancer suggest large chromosomal aberrations, as opposed to focal amplifications, as the dominant driving force for CNV alterations.^21^ Conceptually, we prioritized an outcomes-first approach to model development. We use the term reporter in lieu of gene alteration (including gene gain or gene loss) because the approach is agnostic to the biological causality of specific genes on outcomes. It does not discriminate that causative drivers might be neighboring lesions or aggregate phenomenon rather than the reporter identified. For every gene, 2 reporters were initially assessed: one as a gain and one as a loss. The univariable association with OS was assessed with Cox proportional hazards (PH) models per reporter (gain, loss separately).

To understand the rate and tendencies of false discovery in this data set prior to model building, we evaluated OS associations per reporter separately but with the GISTIC CNV gene calls scrambled within each patient (ie, the GISTIC value for gene X became the value for gene Y, what was Y became Z, randomly, for all genes, per patient). The locations of highly statistically significant alterations in the genome were compared between real and scrambled data.

The distribution of *P* values generated from the univariable OS assessments of reporters from the scrambled data was used to create a cutoff (above upper 99% CI of −log_10_ of *P* values). These reporters were subsequently binned by genomic location, and the reporter at the peak univariable association per region was chosen for the final Cox PH model without further forward or backward feature selection. Orthogonally, LASSO regression^22^ was used as a complementary technique, and the results were compared. The statistical significance threshold was set at *P* < .01.

For internal (within data set) validation of the chosen reporters, the CNV Cox PH model was subjected to bootstrapping based on 90:10 (train:test) partitions and 10 000 iterations of resampling with replacement. During each iteration, a model was built with the aforementioned reporters using the training data, which was used to predict a risk score for the held-out test data. The mean risk score per patient from these iterations of held-out data was used in all subsequent univariable and multivariable assessments of the risk score. Leave-one-out cross validation (LOOCV) was used as a secondary technique for internal validation and results were compared.

#### Model Evaluation

The absolute survival estimate per range of risk score was assessed with the Kaplan-Meier method. The discriminatory ability at specific time points was assessed with the area under the curve (AUC) of time-dependent receiver operating characteristic (ROC) curves. The additive and independent value for prognostication of relative hazards of death relative to existing clinicopathological features in the TCGA database were assessed with a multivariable Cox PH model constructed stepwise. Considered for the model were clinicopathological features with entries available for more than 80% of patients in the TCGA data set with *P* < .05 univariable association with overall survival. Only clinical stage, age, and risk score met these criteria and were included in the model.

#### Gene Ontology

The incidence of molecular function ontologies associated with gene alterations identified within vs not within regions of enriched survival associations was evaluated. The molecular function associated with gene alterations enriched in survival-associated regions was evaluated. Genes were scored using the curated Kyoto Encyclopedia of Genes and Genomes Pathway Database.

#### Software

R software version 3.6.1 (R Project for Statistical Computing) was used for statistical analyses and data visualization with the following packages: survival, survminer, survivalROC, glmnet, biomaRt, ggplot2, gridExtra, clusterProfiler, and forestmodel. KNIME software version 4.0.2 (KNIME AG) was used for data wrangling. Statistical analysis was performed from April to July 2020.

## Results

Among 564 patients with serous ovarian cancer, 34 (6%) identified as Black or African American. The mean (SD) age was 59.7 (11.5) years.

### Global CNV and OS

Prior to evaluation of signatures, we evaluated whether a simple measure, percentage of genes with CNV, was a prognostic feature. Percentage of CNV was not associated with survival (eFigure 2 in the Supplement).

### Model Building

We sought to evaluate the relative risk associated with independent CNV changes observed in ovarian cancer. Scrambled data simulations determined the upper 99th confidence limit −log_10_(*P* value) (ie, 99.5th percentile) to be 2.35, and thus we limited our investigation to reporters with −log_10_(*P* value) > 2.35.

**Figure 1.**
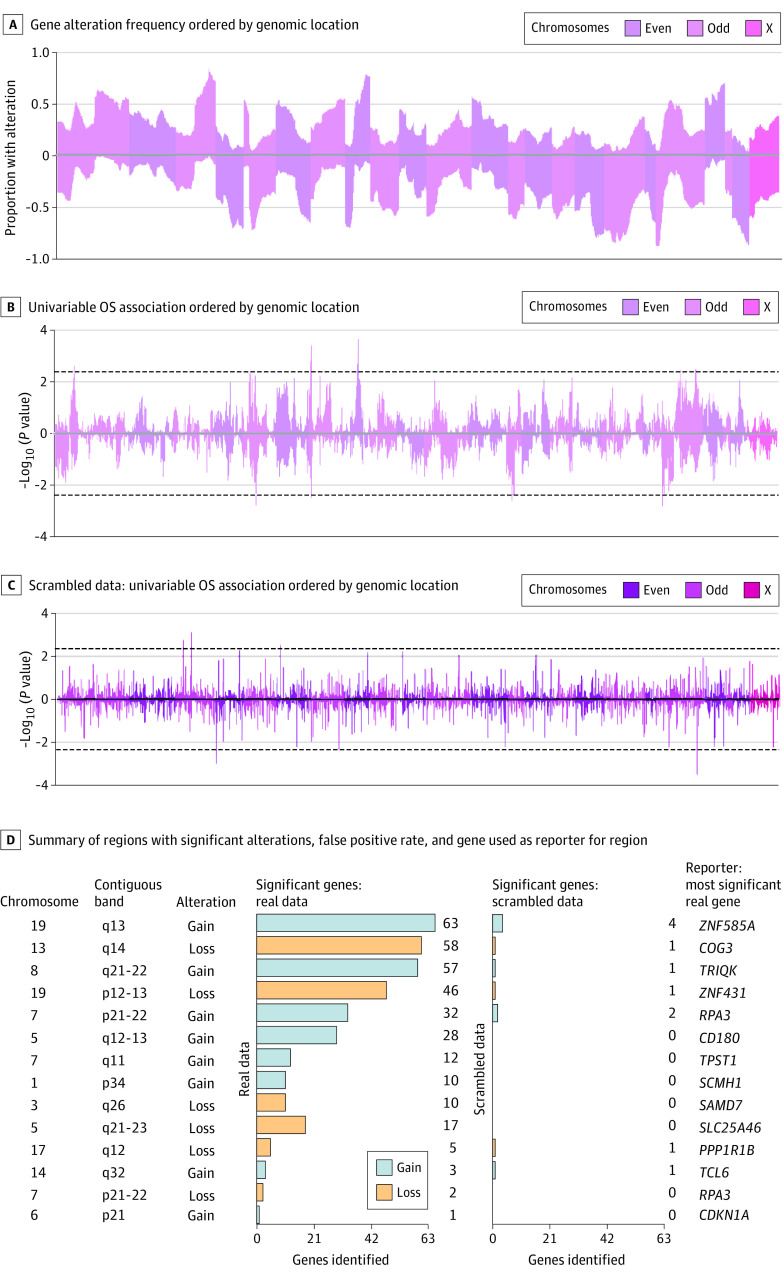
Gene Variation Associated With Overall Survival OS indicates overall survival.

The frequency of alterations (gains or losses) observed was evaluated on a gene by gene basis across the entire genome. We measured an accompanying univariable association with overall survival per reporter: gene gain or loss (Figure 1A and Figure 1B). The reporters that were significantly associated with overall survival (OS) showed an enrichment in islands of adjacent genes. We identified 13 regions of clustered significance. Among these, only 7q22 was bilaterally associated with OS: both gains and losses were significant. In contrast, an analysis of OS associations with scrambled data did not display clustered associations (Figure 1C and Figure 1D).

**Figure 2.**
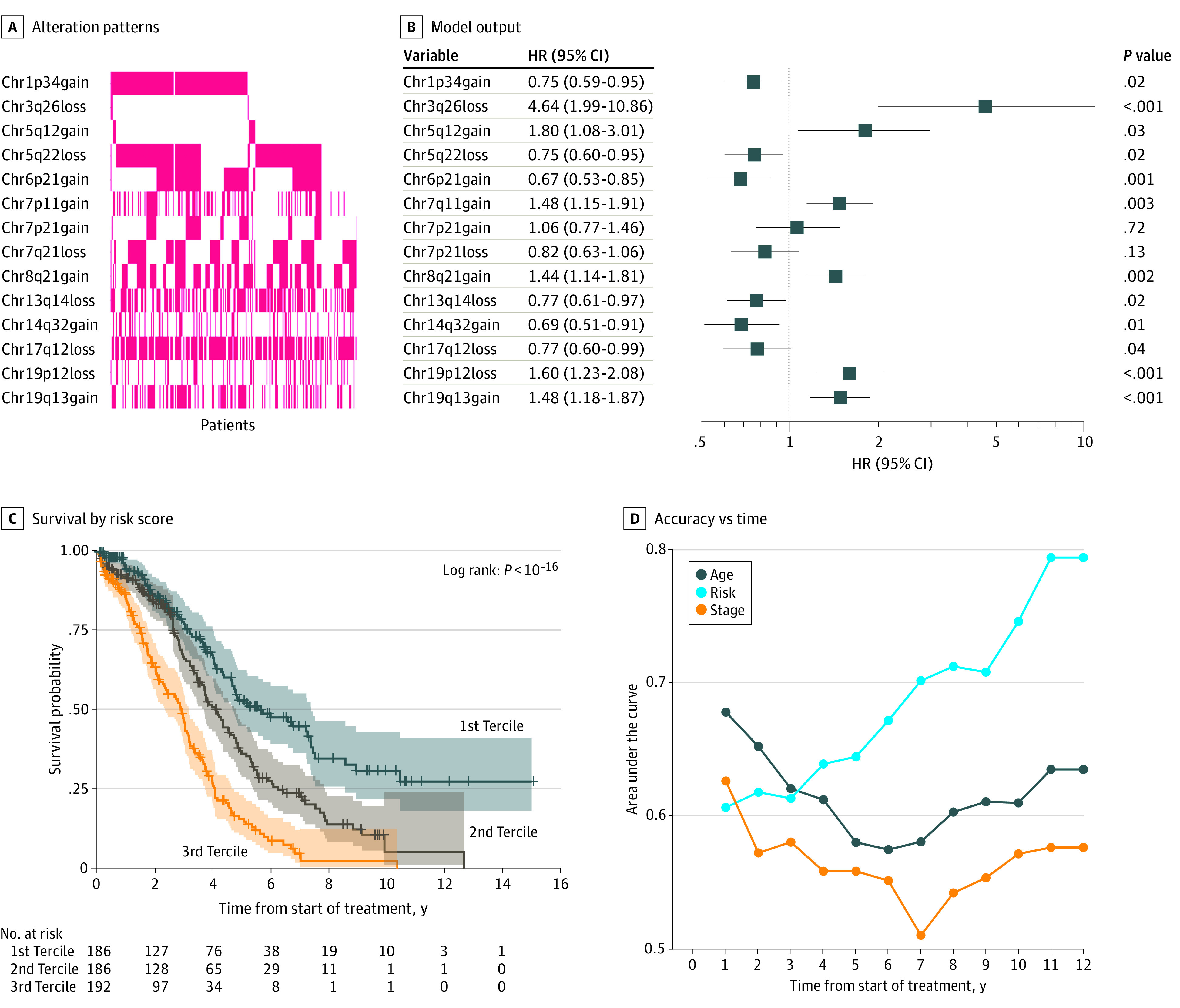
Copy Number Variation Associated With Cox PH Model HR indicates hazard ratio.

Orthogonal use of LASSO regression to derive the most important reporters chose 13 of the top 14 reporters from the same regions determined by the clustered approach (eTable 1 in the Supplement). Each of the identified 13 regions had many more significant alterations than the scrambled data (Figure 1D). The identified reporters displayed a high degree of independence in their incidence (Figure 2A) as well as additive and independent contributions to the Cox PH model (Figure 2B).

### Univariable Bootstrapped Model Performance

The output from models containing these features (Figure 2B) were next bootstrapped (with replacement) 10 000 times to adjust for optimism bias, and used to generate an internally validated risk score. Use of LOOCV revealed almost identical results (eFigure 4 in the Supplement). Binning the risk score into terciles yielded 3 readily distinguishable risk groups; the highest risk group having a median survival of 2.9 years (95% CI, 2.3-3.2 years) compared to a median survival of the low-risk group of 5.7 years (95% CI, 4.7-7.4 years). Considering only the 5-year time point, Kaplan-Meier estimates of survival were 15% (95% CI, 10%-22%) and 53% (45%-62%), respectively (Figure 2C). When assessed as a continuous variable, the univariable discriminatory power at specific points in time was compared with patient age and clinical stage (Figure 2D). Unlike these clinical features, which remain relatively constant, the discriminatory power increases with respect to time. However, it should be noted that, in part owing to the relative nature of disease, there are few patients available for assessment at later time points as indicated (Figure 2C). At the 10-year time point, the CNV risk score’s AUC was 0.747 (Figure 2D).

### Multivariable Bootstrapped Model Performance

**Figure 3.**
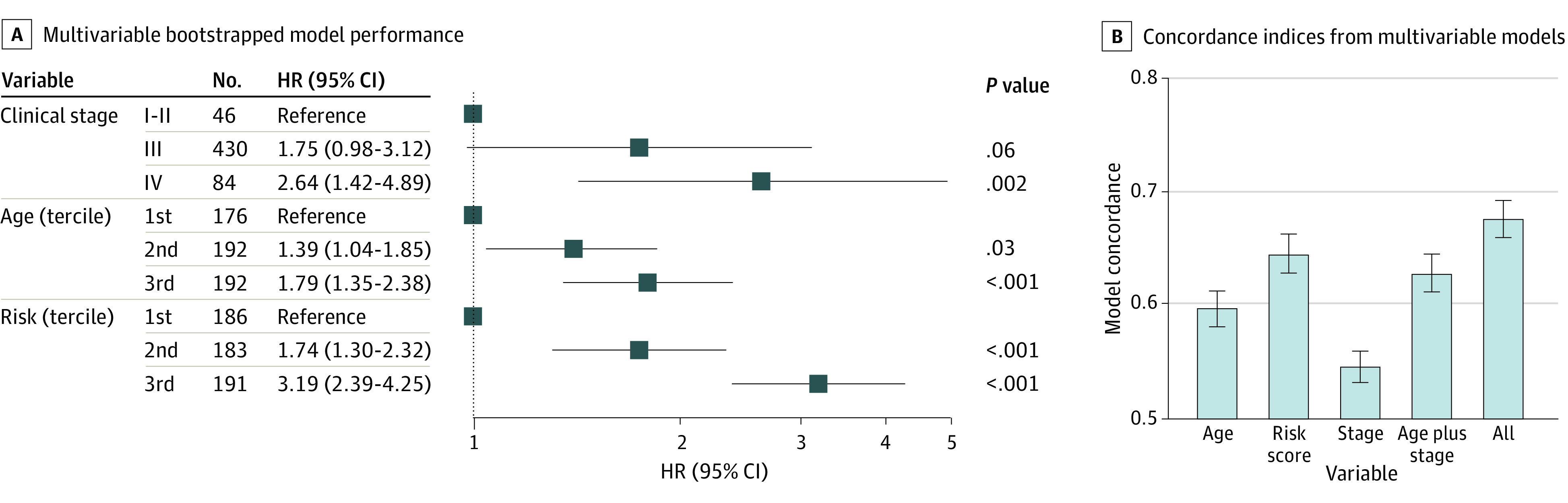
Comparison of Hazard Ratio (HR) by Risk Score, Age, and Clinical Stage

The bootstrapped risk score was evaluated as part of a multivariable Cox PH model in conjunction with age and clinical stage, with additive and independent associations (Figure 3A). Age and the risk score were included in the model as terciles for easier comparison with the clinical stage. Separately, models constructed including age and risk as continuous variables, along with clinical stage, indicate that the risk score alone provided more discriminatory power than both age and clinical stage combined to prognosticate overall survival, and combining the risk score with these features substantially adds to the models (*P* < .001) (Figure 3B). Sensitivity analyses additionally including grade and race into alternate model builds showed consistent results (eFigure 5 in the Supplement).

### Comparison With Alteration Burden, *BRCA* Status, and Genomic Scar Signatures

**Figure 4.**
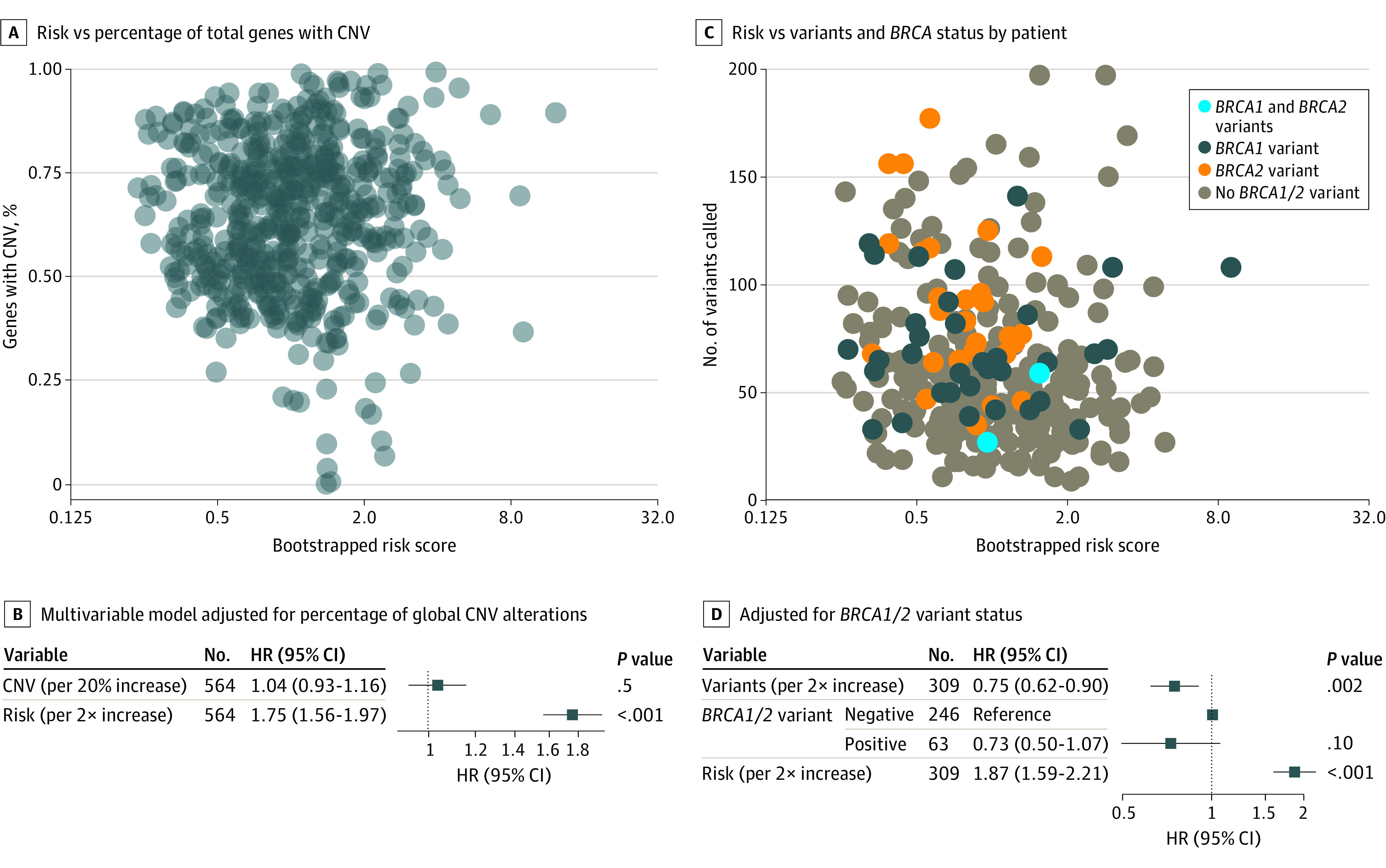
Cox Proportionate Hazard as a Function of Copy Number Variations (CNV) or *BRCA* Status HR indicates hazard ratio.

Total genes in the TCGA database with copy number alterations (GISTIC call not equal to 0) was not associated with risk score (Figure 4A) and risk score strength was not diminished in a multivariable model when adjusted for percentage of global CNV alterations (Figure 4B). The risk score was not associated with total mutational burden or *BRCA1/2* status (Figure 4C). However, the total variations reported per patient was additive and independent to the risk score to prognosticate OS, even when adjusted for by *BRCA1/2* mutational status (Figure 4D).

Patterns of copy number variations as a proxy for genomic scars from DNA repair deficiencies, both proprietary and not, have been investigated as potential predictive biomarkers for PARPi benefit,^2,4^ as opposed to a general prognostic signature. Open source signatures include: number of discrete chromosomal lesions including telomeric imbalances (NtAI), large scale transitions (LST), and homologous recombination deficiency score loss of heterozygosity (HRD-LOH).^21^ The risk score was not associated with these genomic scar signatures (eFigure 6 in the Supplement), and had stronger associations with survival than these measures.

### Biological Mechanism Associations

Although biological evaluation was not a central focus of this work, we surveyed the known biology of genes (gene ontology) surrounding the identified reporters, as these are prime candidates for future, deeper investigations. In terms of total enrichment within the 13 regions significantly associated with survival, the most significant ontology was mRNA binding involved in posttranscriptional gene silencing (eTable 2 in the Supplement) encompassing 21 miRNAs. Protein kinase regulator activity, an ontology term, was the enriched term most distributed across the regions, present in 6 of 13 regions encompassing the following genes: *CCNB1, APC, CDKN1A, PKIA, TCL1B, TCL1A, *and* PPP1R1B*.

### Correlation With Commercially Available Multigene Assays

There now exist FDA-approved, commercially available multigene assays that can be ordered by physicians. These assays all include copy number evaluations, but do not evaluate all of the genes in the TCGA database. However, of the identified 13 regions (14 alterations), there exist genes on the assay for 12, and 11, of these regions among the MSK-IMPACT, and FoundationOne CDx assays, respectively (eTable 3 in the Supplement). We reevaluated the resulting change in model performance using the available reporters with each assay. The CNV risk score has a bootstrapped concordance (SE) of 0.644 (0.014). The assays had respective resulting concordance (SE) indices of 0.623 (0.018) and 0.626 (0.018).

## Discussion

Prognostication of patient survival in ovarian cancer does not widely use additive genomic features. Using the TCGA database, we sought to develop a risk score that might be complementary to existing nomograms. We chose to focus on copy number variations, because of the dynamic range observed in OVCA, diagnostic considerations, and the availability and ease of potential incorporation to existing commercially available, FDA-approved multigene assays. The nominal drop in discriminative ability using an assay repurpose approach suggests that these tests could potentially be used to prognosticate patient risk.

The ideal comparison of the additive discriminatory power of the CNV risk score would be in conjunction with established clinical features used in current nomograms for ovarian cancer survival, and this would be a logical future extension of the base model presented here. A summary of such models and their contributing clinicopathological features, as well as the availability such features in the TCGA HGSOC firehose database is provided in the Table. Although age, tumor grade, and stage are sufficiently available in the TCGA database, other commonly used features such as preoperative albumin, Ashkenazi ancestry, and maximum diameter of residual tumor (ie, degree of success of surgical resection) were not. Nomograms built from different cohorts do not yield a consistent set of clinical features chosen for final models from cohort to cohort.^7,8,9,23^ One possible contributing factor might be the (unavoidable) subjective nature of some clinical assessments. However, one of the most consistent strong features observed among these nomograms is the completeness of initial surgery^7,8,9,24^ (Table), which had insufficient completeness in our data set (9.9%) for comparison. Extending the genetic model to a formal nomogram construction with the CNV risk score would be premature lacking these data. Nonetheless, we felt it could be informative to assess the additive and independent discriminatory value of the CNV risk score in comparison to age, clinical stage, pathological grade, and race.

**Table.** Prognostic Nomograms for Overall Survival

Data collected	TCGA database	Barlin et al, 2011^7^	Rutten et al, 2014^9^	Xu et al, 2017^8^	Hamilton et al, 2018^24^
Data source	OVCA Firehose accessed July 2020 (%)	One-site prospective from MSKCC (n = 478)	Retrospective three center (n = 840)	SEER (n = 10 692)	Original collected data (n = 3010)
Reported nomogram OS performance	NA	C-index: 0.714	C-index: 0.71 (95% CI, 0.69- 0.74)	C-index: 0.757 (95% CI, 0.741-0.773)	10-y AUC: 0.729
Dates of initial treatment	1992-2013	1996-2004	1998-2010	2004-2013	Not reported
Age	Yes (100%)	Yes	Yes	Yes	No
FIGO tumor grade	Yes (99.3%)	Yes	Yes	Yes	No
Preoperative albumin, g/dL	No	Yes	No	No	No
Max diameter residual tumor	No	Yes	Yes	No	Yes
Histological subtype	Yes (100%; all serous)	Yes	Yes	No	No
ASA score	No	Yes	No	No	No
Breast/ovarian hereditary cancer syndrome	Yes (4.1%)	Yes	Yes	No	No
Race	Yes (94.7%)	No	No	Yes	No
Marital status	No	No	No	Yes	No
Tumor location	Yes (100)	No	No	Yes	No
Clinical stage	Yes (99.1)	No	No	Yes	No
LODDS	No	No	No	Yes	No
Primary vs interval surgery	No	No	Yes	No	No
Performance status	Yes (33.4%)	No	No	No	No
Amount of ascites	No	No	No	No	Yes
CA-125 level	No	No	No	No	Yes

Abbreviations: ASA, American Society of Anesthesiologists; AUC, area under the curve; CA-125, Cancer antigen 125; C-index, concordance index; FIGO, The International Federation of Gynecology and Obstetrics; MSKCC, Memorial Sloan Kettering Cancer Center; LODDS, log of odds of #pos:#neg lymph nodes; OS, overall survival; OVCA, ovarian carcinoma; SEER, Surveillance, Epidemiology, and End Results.

The observed increase in CNV risk score discriminatory ability over time (Figure 2D) is consistent with the notion that with increasing time, it is the biology of the tumor that determines outcome. This phenomenon has also been observed in gene expression prognostic signatures.^14^ At the 10-year time point, the CNV risk score had an AUC of 0.747. In comparison, Hamilton and colleagues reported a model containing residual tumor burden, CA-125 level, and amount of ascites, with a 10-year ROC AUC of 0.729.^24^ A suitable cohort combining these features might better predict long-term survivors.

All samples in the TCGA database were collected prior to initiation of systemic therapy, representing a prefirst-line time point. Most patients likely had a combination platinum-taxane chemotherapy as part of their therapeutic regimen, which exploits DNA repair deficiencies. However, with the exception of NBN at 8q21, DNA repair genes were notably absent from regions in the signature.

Several chromosomal alterations shown to be associated with increased risk have been described previously, including regions on chromosome 3, 5 and 7.^25,26^ The 2 regions on chromosome 19 are noteworthy as they represent previously unrecognized regions associated with ovarian cancer malignancy, although they have been associated with lung, prostate, and breast cancer.^27,28,29^ Specifically, the miRNA cluster on Chr19q has been implicated as a driver in triple negative breast cancer.^29^ Genes involved in epithelial to mesenchymal transition (TGFB1 and LTBP4) are present in this region as well. It should be noted that for a feature to be prognostic, it must be altered in some patients and not altered in others. A feature altered in the vast majority, or conversely, almost no patients, would be less likely to have strong prognostic associations, and less likely to arrive in a CNV prognostic signature.

The identified regions could be added to lists of inquiry for drug development that might be currently underappreciated. The survival curves from the patients in the highest at-risk tercile are consistent with modest effect of initial therapy, highlighting the importance of novel drug development and opportunities for improved molecular disease characterization. Conversely, the first tercile may include a cadre of patients that experience prolonged disease remission or are potentially cured.

### Limitations and Strengths

Limitations of this study included insufficient clinical features for formal nomogram comparisons, lack of validation with external data sets, serous pathology–only data set, and disproportionate patients in the TCGA database who were self-reported White or European ancestry (82%). Also, the clinical-genomic-outcome associations represent the standard of care from 1992 to 2013; there have been substantial advancements in adjuvant therapy over time, with PARP inhibitors now approved in the front line and recurrent settings as a maintenance and treatment strategy.^2,3,4,5,6^ Platinum and taxane therapy was the prominent systemic treatment for patients treated in the era collected for the TCGA database and is unlikely to be completely supplanted in the near future.

This study also had several strengths. These included the objectivity and importance of the end point (overall survival), the relative simplicity of the model compared with previous signature work, preanalytic stability of the analyte (CNV) and applicability of the findings, given potential low consumable and bioinformatic load for assay development, or repurposement of existing FDA-approved assays.

## Conclusions

This genetic association study found that a CNV-based risk score is independent to and stronger than open-source ovarian cancer genomic biomarkers to prognosticate OS. External validation, especially evaluated in concert with robust clinical features, could provide rapidly accessible additional patient prognostication. Identified genomic regions most tied to OS could inform drug discovery efforts.

## Notes

Supplement. **eFigure 1.** Analysis Flowchart **eFigure 2.** Association of Survival with Proportion of Genes with CNV **eFigure 3.** CNV Plots and Clustered Regions by Risk Score Tercile **eFigure 4.** Bootstrapping Internal Validation Comparison to LOOCV Internal Validation **eFigure 5.** Alternate Cox PH Models **eFigure 6.** Comparison of Risk Score to Genomic Scar Signatures **eTable 1.** Comparison of LASSO-Chosen Features vs the Main Model Features and Performance **eTable 2.** FDA-Approved Panel Reporter Approximations **eTable 3.** How to Use Risk Score
