# Errors in Figure Captions

**DOI:** 10.1001/jamanetworkopen.2021.25709

**Published:** 2021-08-11

In the Original Investigation titled “Association of Copy Number Variation Signature and Survival in Patients With Serous Ovarian Cancer,”^1^ published June 28, 2021, the captions were missing for Figure 1, Figure 2, Figure 3, and Figure 4. This article has been corrected.^1^
