# Supplementary material for: Association of Copy Number Variation Signature and Survival in Patients With Serous Ovarian Cancer
Source: JAMA Netw Open. 2021 Jun 28;4(6):e2114162. doi: 10.1001/jamanetworkopen.2021.14162 (PMC8239953; doi:10.1001/jamanetworkopen.2021.14162)
Supplement: Supplement. — eFigure 1. Analysis Flowchart eFigure 2. Association of Survival with Proportion of Genes with CNV eFigure 3. CNV Plots and Clustered Regions by Risk Score Tercile eFigure 4. Bootstrapping Internal Validation Comparison to LOOCV Internal Validation eFigure 5. Alternate Cox PH Models eFigure 6. Comparison of Risk Score to Genomic Scar Signatures eTable 1. Comparison of LASSO-Chosen Features vs the Main Model Features and Performance eTable 2. FDA-Approved Panel Reporter Approximations eTable 3. How to Use Risk Score [file jamanetwopen-e2114162-s001.pdf]

Supplemental Online Content

Graf RP, Eskander R, Brueggeman L, Stupack DG. Association of copy number variation signature and survival in patients with serous ovarian cancer. *JAMA Netw Open.* 2021;4(6):e2114162. doi:10.1001/jamanetworkopen.2021.14162

eFigure 1. Analysis Flowchart

eFigure 2. Association of Survival with Proportion of Genes with CNV

eFigure 3. CNV Plots and Clustered Regions by Risk Score Tercile

eFigure 4. Bootstrapping Internal Validation Comparison to LOOCV Internal Validation

eFigure 5. Alternate Cox PH Models

eFigure 6. Comparison of Risk Score to Genomic Scar Signatures

eTable 1. Comparison of LASSO-Chosen Features vs the Main Model Features and Performance

eTable 2. FDA-Approved Panel Reporter Approximations

eTable 3. How to Use Risk Score

This supplemental material has been provided by the authors to give readers additional information about their work.

© 2021 Graf RP et al. *JAMA Network Open.*

eFigure 1: Analysis Flowchart

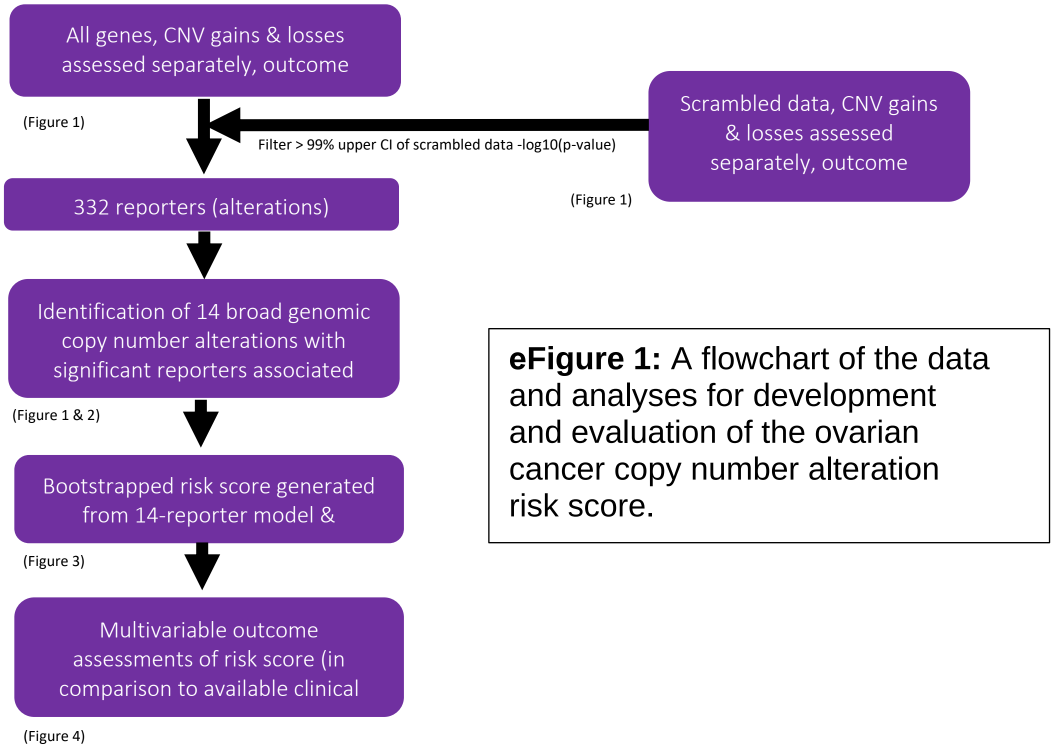

eFigure 1: A flowchart of the data and analyses for development and evaluation of the ovarian cancer copy number alteration risk score.

eFigure 2:
Association of Survival with Proportion of Genes with CNV

A

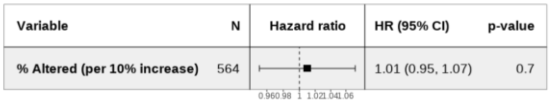

eFigure 2: (A) Univariable Cox PH model evaluating the relationship between the % of genes in genome with copy number variations (GISTIC score not equal to 0) and overall survival.
(B) Kaplan-Meier estimations of overall survival by % of genes in genome with copy number variations, binned into terciles.

B

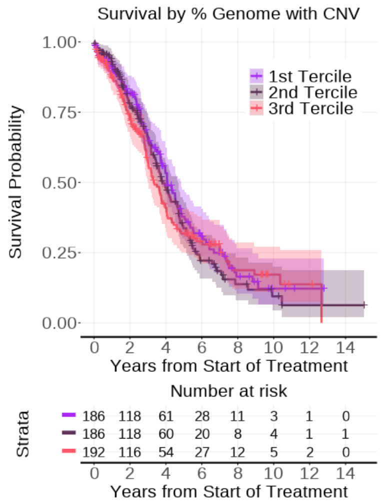

© 2021 Graf RP et al. *JAMA Network Open.*

eFigure 3: CNV Plots and Clustered Regions by Risk Score Tercile

A

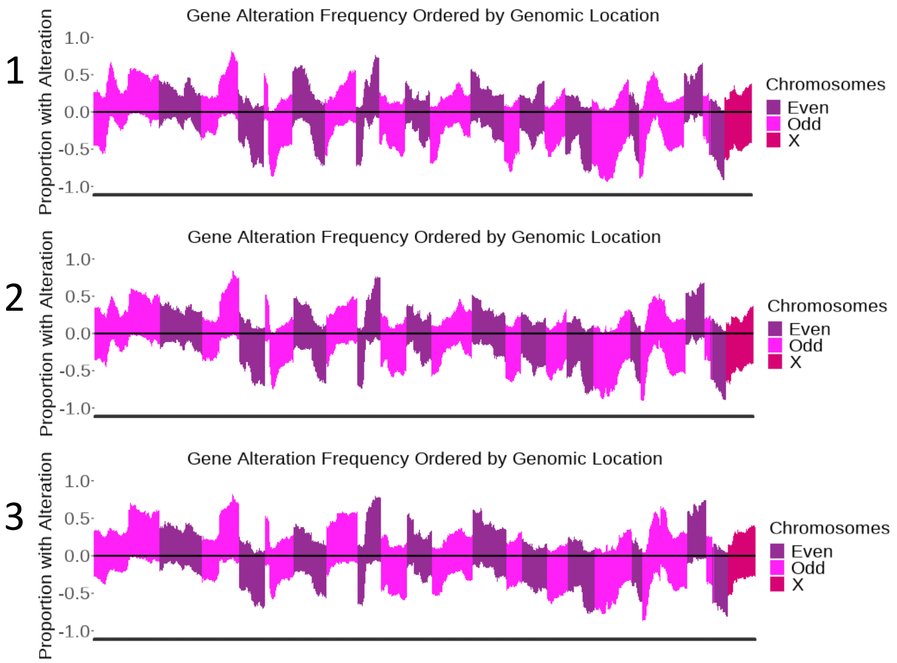

B

TERCILE

Region	Alteration	1	2	3
1p34	gain	0.67	0.49	0.36
3q26	loss	0	0	0.03
5q12	gain	0	0.01	0.11
5q22	loss	0.75	0.59	0.38
6p21	gain	0.6	0.42	0.3
7p21	gain	0.11	0.22	0.39
7p21	loss	0.58	0.43	0.24
7q11	gain	0.19	0.39	0.61
8q21	gain	0.39	0.54	0.71
13q14	loss	0.81	0.67	0.39
14q32	gain	0.39	0.16	0.09
17q12	loss	0.9	0.72	0.58
19p12	loss	0.05	0.21	0.34
19q13	gain	0.24	0.42	0.66

Poorer Outcome
Improved Outcome

eFigure 3: Genomic alterations plots are shown (A) by first, second and third tercile (labeled 1, 2, 3). Frequency of gains (either GISTIC +1 or +2) indicated as the height above center line, frequency of losses (either GISTIC -1 or -2) indicated as depth below center line (negative values). Genes ordered by location in the genome. (B) The specific locations of clustered regions associated with survival included in the risk score and associated incidence of alteration within each tercile.

© 2021 Graf RP et al. *JAMA Network Open*.

eFigure 4:
Bootstrapping Internal Validation Comparison to LOOCV Internal Validation

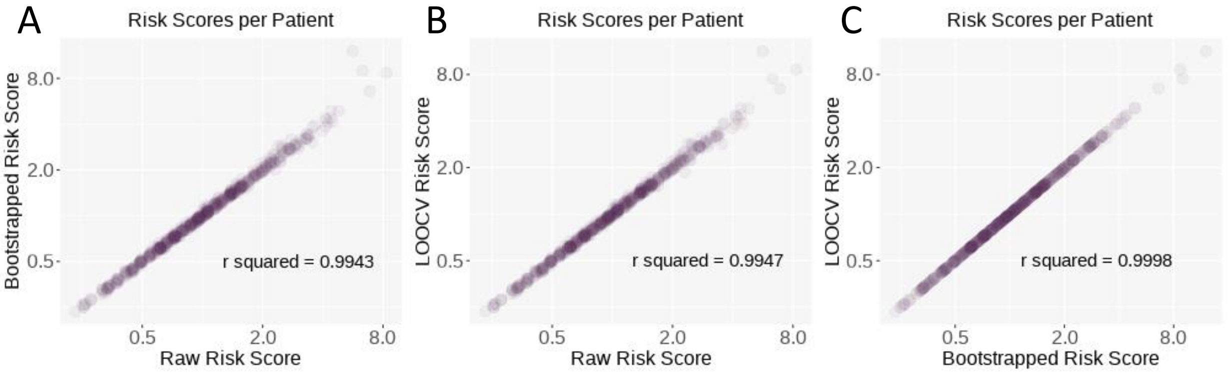

eFigure 4: Scatter plots comparing the risk score (A) raw vs. bootstrapped, (B) raw vs. leave one out cross-validation (LOOCV), and (C) bootstrapped vs. LOOCV. Coefficient of determination is shown from a linear model incorporating compared values per graph.

© 2021 Graf RP et al. *JAMA Network Open.*

eFigure 5: Alternate Cox PH Models

A

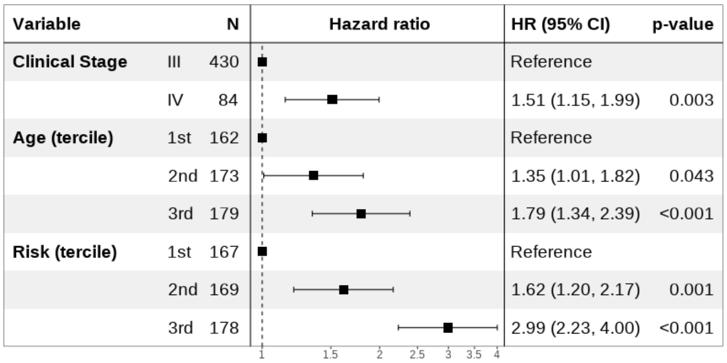

Variable		N	HR (95% CI)	p-value
**Clinical Stage**	III	430	Reference	
	IV	84	1.51 (1.15, 1.99)	0.003
**Age (tercile)**	1st	162	Reference	
	2nd	173	1.35 (1.01, 1.82)	0.043
	3rd	179	1.79 (1.34, 2.39)	<0.001
**Risk (tercile)**	1st	167	Reference	
	2nd	169	1.62 (1.20, 2.17)	0.001
	3rd	178	2.99 (2.23, 4.00)	<0.001

B

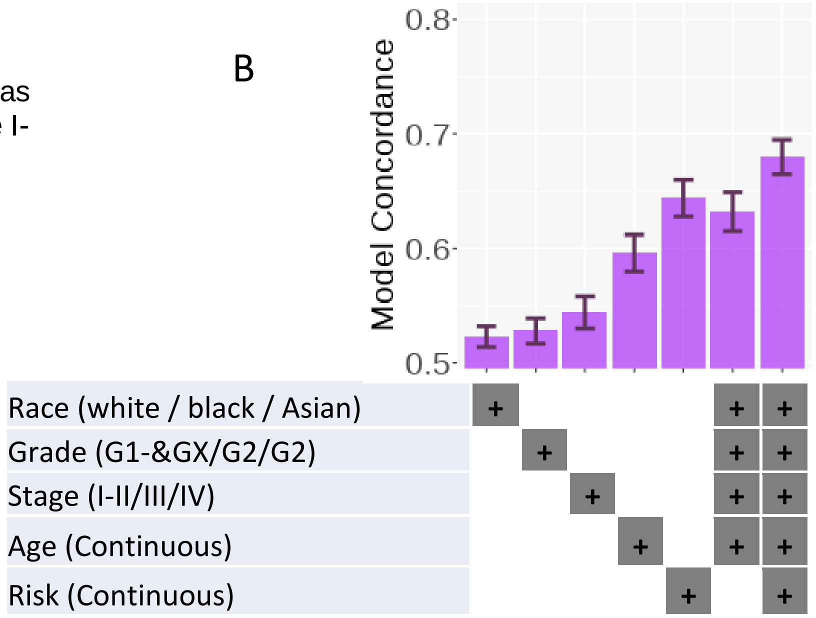

eFigure 5:
(A) Multivariable model as per Figure 4, with Stage I-II patients excluded and (B) Figure 4 model comparisons with expanded features and continuous variables.

© 2021 Graf RP et al. *JAMA Network Open*.

eFigure 6: Comparison of Risk Score to *Genomic Scar* Signatures

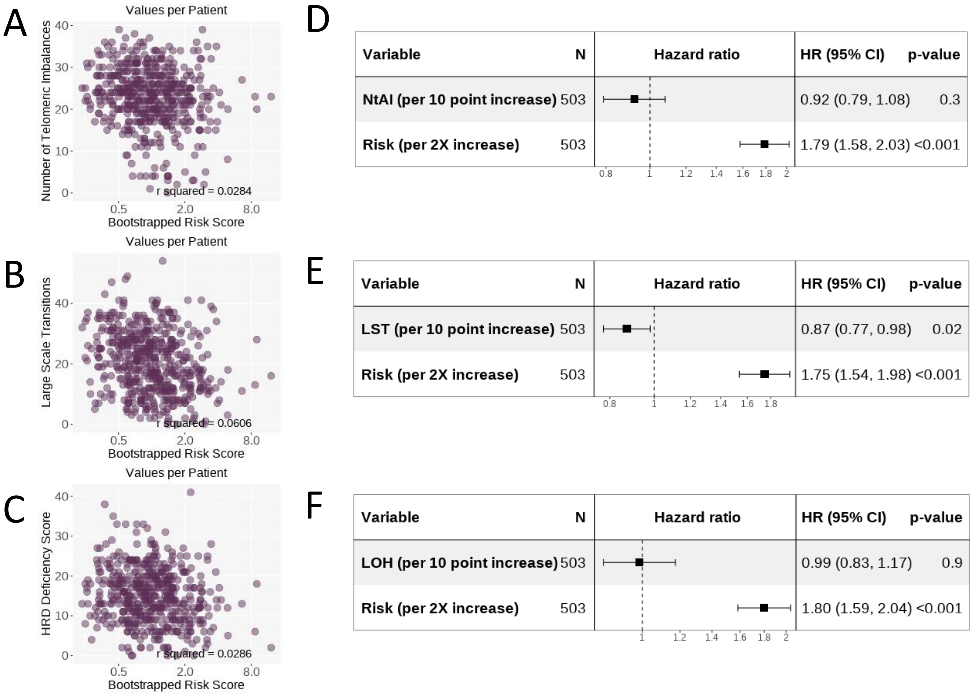

<u>eFigure 6</u>: Scatter plots showing relationship between Bootstrapped Risk Score and genomic scar signatures (A) Number of telomeric imbalances (NtAI), (B) large scale transitions (LST) and (C) homologous recombination deficiency score loss of heterozygosity (LOH). Multivariable models incorporating risk score and (D) NtAI, (E) LST, (F) LOH. NtAI, LST, and LOH values were obtained from reported supplementary data from Marquard et al 2015. Patients without reported values were excluded from analyses.

© 2021 Graf RP et al. *JAMA Network Open.*

eTable 1:
Comparison of LASSO-chosen features vs. the main model features and performance

A

Chromosome	Band	Alteration	Abs Coefficient	Reporter Chosen by LASSO Model
8	q21	gain	0.0873	RNA5SP273
1	p34	gain	0.0818	SCMH1
19	p13	loss	0.0738	RDH8
7	q11	gain	0.0543	TPST1
5	q23	loss	0.0517	DMXL1
13	q14	loss	0.0410	COG3
7	p21	loss	0.0389	RPA3
19	q13	gain	0.0368	ZNF585A
6	p21	gain	0.0267	CDKN1A
5	q22	loss	0.0154	SLC25A46
6	p22	gain	0.0139	RNA5SP205
8	q22	gain	0.0014	TRIQK
7	p22	gain	0.0013	ZDHHC4
6	q14	loss	0.0001	PGM3

B

	Main Cox Model	LASSO-chosen features in Cox Model
Reporters	14	14
Unadjusted concordance	0.662 (0.016)	0.655 (0.016)
Bootstrapped concordance	0.644 (0.016)	0.634 (0.017)
LOOCV concordance	0.644 (0.016)	0.634 (0.017)

eTable 1: (A) Top 14 reporters chosen by the LASSO model. Reporters in same regions chosen by main model are colored **purple**, and reporters identical to ones chosen in the main model are colored **red**. (B) comparison of main model vs. Cox model constructed with LASSO-chosen features: concordance, raw vs. internally validated. LOOCV = “leave on out cross-validation.”

© 2021 Graf RP et al. *JAMA Network Open.*

eTable 2: FDA-Approved Panel Reporter Approximations

Chromosome	Contiguous Band	Alteration	Reporter used in CNV Risk Score model:	Closest Reporter: MSK-IMPACT	Closest Reporter: FoundationOne CDx
1	p34	Gain	SCMH1	MYCL	MYCL
3	q26	Loss	SAMD7	PRKCI	PRKCI
5	q12-13	Gain	CD180	PIK3R1	PIK3R1
5	q21-23	Loss	SLC25A46	APC	APC
6	p21	Gain	CDKN1A	CDKN1A	CDKN1A
7	p21-22	Gain	RPA3	PMS2	PMS2
7	p21-22	Loss	RPA3	PMS2	PMS2
7	q11	Gain	TPST1	n/a	n/a
8	q21-22	Gain	TRIQK	NBN	NBN
13	q14	Loss	COG3	CYSLTR2	RB1
14	q32	Gain	TCL6	DICER1	n/a
17	q12	Loss	PPP1R1B	ERBB2	ERBB2
19	p12-13	Loss	ZNF431	DNMT1	SMARCA4
19	q13	Gain	ZNF585B	AKT2	AKT2
Model bootstrapped concordance:			0.644 (0.014)	0.623 (0.018)	0.626 (0.018)

eTable 2: Table of regions with highest amount of significant reporters, associated reporter used in the base model, and the closest reporter in FDA-cleared panels, as well as the resulting within-TCGA internally validated concordance index from models with indicated reporters.

© 2021 Graf RP et al. *JAMA Network Open.*

eTable 3: How To Use Risk Score

A $relative\ patient\ risk = e\text{^}((\Sigma \text{ coefficients}) - 0.323)$

B

Chr	Contiguous Band	Alteration	Reporter Gene	Coefficient
1	p34	Gain	SCMH1	-0.289
3	q26	Loss	SAMD7	1.535
5	q12-13	Gain	CD180	0.587
5	q21-23	Loss	SLC25A46	-0.281
6	p21	Gain	CDKN1A	-0.397
7	q11	Gain	TPST1	0.392
7	p21-22	Gain	RPA3	0.058
7	p21-22	Loss	RPA3	-0.199
8	q21-22	Gain	TRIQK	0.362
13	q14	Loss	COG3	-0.262
14	q32	Gain	TCL6	-0.378
17	q12	Loss	PPP1R1B	-0.261
19	p12-13	Loss	ZNF431	0.470
19	q13	Gain	ZNF585B	0.395

C

Chr	Contiguous Band	Alteration	Reporter Gene	Coefficient	Has alteration? 1 = yes, 0 = no	Coefficients to sum
1	p34	Gain	SCMH1	-0.289	1	-0.289
3	q26	Loss	SAMD7	1.535	0	0
5	q12-13	Gain	CD180	0.587	1	0.587
5	q21-23	Loss	SLC25A46	-0.281	0	0
6	p21	Gain	CDKN1A	-0.397	0	0
7	q11	Gain	TPST1	0.392	1	0.392
7	p21-22	Gain	RPA3	0.058	0	0
7	p21-22	Loss	RPA3	-0.199	0	0
8	q21-22	Gain	TRIQK	0.362	1	0.362
13	q14	Loss	COG3	-0.262	1	-0.262
14	q32	Gain	TCL6	-0.378	0	0
17	q12	Loss	PPP1R1B	-0.261	0	0
19	p12-13	Loss	ZNF431	0.470	0	0
19	q13	Gain	ZNF585B	0.395	1	0.395
Sum of coefficients for hypothetical Patient A:						1.185
Relative patient risk:					e^(0.203 – 1.185)	
Relative patient risk:					2.67	

D

Tercile	Risk Score	Median Years OS (95% CI)
1st	< 0.72	5.7 (4.7 - 7.4)
2nd	0.73 to 1.35	4.1 (3.7 - 4.8)
3rd	> 1.36	2.9 (2.3 - 3.2)

eTable 3: (A) Relative patient risk equation. (B) Summary of final model components and coefficients, trained on the entire TCGA dataset. (C) Example application of risk score to hypothetical Patient A. Patient A has gain of SCMH1, gain of CD180, gain of TPST1, gain of TRIQK, loss of COG3, gain of ZNF585B, but not the other indicated alterations in the model. Interpretation: Considering only copy number alterations, Patient A is anticipated to have a 2.67X higher likelihood of dying sooner than the average patient in the TCGA firehose database (if given similar treatment to patients contributing to the TCGA database). (D) Risk score ranges shown by tercile and OS for patients in TCGA cohort, for comparison.

© 2021 Graf RP et al. *JAMA Network Open*.
